# Prognostic value of the lactate dehydrogenase to albumin ratio in advanced non-small cell lung cancer patients treated with the first-line PD-1 checkpoint inhibitors combined with chemotherapy

**DOI:** 10.3389/fimmu.2025.1473962

**Published:** 2025-02-12

**Authors:** Meifeng Luo, Huiting Wei, Moqin Qiu, Cuiyun Su, Ruiling Ning, Shaozhang Zhou

**Affiliations:** Department of Respiratory Oncology, Guangxi Medical University Cancer Hospital, Nanning, China

**Keywords:** LAR, prognosis, non-small cell lung cancer, PD-1 checkpoint inhibitors, chemotherapy

## Abstract

**Background:**

This study aimed to investigate the prognostic value of pretreatment lactate dehydrogenase to albumin ratio (LAR) in advanced non-small cell lung cancer (NSCLC) patients treated with first-line programmed cell death protein 1 (PD-1) checkpoint inhibitors and chemotherapy.

**Methods:**

A retrospective cohort study was conducted on advanced NSCLC patients treated with first-line PD-1 checkpoint inhibitors plus chemotherapy at Guangxi Medical University Cancer Hospital. The receiver operating characteristic (ROC) analysis determined the optimal LAR cutoff values for prediction. Univariate and multivariate analyses identified independent prognostic factors, and survival curves were estimated using the Kaplan-Meier method. Subgroup analysis evaluated the association between high LAR and disease progression and death risk.

**Results:**

A total of 210 patients were enrolled, with a mean age of 58.56 ± 10.61 years and a male proportion of approximately 79.05%. ROC analysis found the optimal LAR cutoff value was 5.0, resulting in a sensitivity of 78.87% and a specificity of 44.6% (area under the ROC curve 0.622; P = 0.001). Multivariate analysis revealed a significant positive association between LAR and overall survival (OS) after adjusting for confounders (HR = 2.22, 95% CI = 1.25-3.96, P = 0.007). Subgroup analysis confirmed the relationship between high LAR and the risk of disease progression and death across all patient subgroups.

**Conclusions:**

Pretreatment LAR may be a potential independent prognostic marker for advanced NSCLC patients receiving PD-1 checkpoint inhibitors plus chemotherapy. A large-scale, prospective study is necessary to confirm these findings.

## 1 Introduction

Lung cancer is an important health issue faced by the global populations (1), with non-small cell lung cancer (NSCLC) remaining the most prevalent type. In recent years, the advent of immune checkpoint inhibitors (ICIs), specifically programmed cell death protein 1 (PD-1) or programmed death-ligand 1 (PD-L1) antibodies, has dramatically altered the therapeutic landscape for NSCLC due to their superior efficacy compared to conventional chemotherapy (2–4). Large-scale clinical trials, such as Keynote 189 (5), Keynote 407 (6), CheckMate-9LA (7) and IMPOWER150 (8), have established anti-PD-1/PD-L1 combined with chemotherapy therapy as the standard of care for advanced NSCLC patients lacking driver mutations. Despite significant progress in the field of immune checkpoint therapy, there remains a critical unmet clinical need for reliable biomarkers capable of accurately predicting treatment efficacy and patient prognosis. The predictive capacity of PD-L1 expression (TPS score), tumor mutational burden (TMB), and gut microbiota continues to be a topic of considerable debate (9, 10). There is an urgent need to develop biomarkers that can accurately forecast the response to immunotherapy, which can assist clinicians in optimizing subsequent treatment strategies.

In immunotherapy research, blood-based biomarkers, including both tumor-derived markers such as blood tumor mutation burden (bTMB) and microRNA, and non-tumor-derived markers like neutrophil-to-lymphocyte ratio (NLR), platelet-to-lymphocyte ratio (PLR), Systemic Immune-Inflammation Index (SII), soluble PD-L1 (sPD-L1), and soluble LAG-3 (sLAG-3), are becoming a focal point of study (11–15). Their popularity is growing due to their ease of collection, non-invasiveness, and their carrying potential capacity to provide information about treatment effectiveness, patient prognosis, and the response and resistance to immune therapy. Prior to this, our research group conducted relevant studies using heat shock protein 90a (HSP90a), revealing its association with poor prognosis in advanced non-small cell lung cancer patients treated with first-line immunotherapy combined with chemotherapy (16). But we found that not all institutions routinely test this indicator. Therefore, simple, cost-effective, and easily scalable biomarkers are needed to monitor the prognosis of patients undergoing immune checkpoint inhibitors (ICIs) treatment.

Lactate dehydrogenase (LDH) is a pivotal glycolytic enzyme, with increased levels signifying inflammation, hypoxia, and necrosis (17, 18). High LDH often correlates with tumor burden and malignancy (19). Albumin (ALB), an essential plasma protein, helps maintain osmotic pressure and transport nutrients, with decreased levels indicative of malnutrition or liver dysfunction (20). The lactate dehydrogenase to albumin ratio (LAR), a systemic inflammatory biomarker, reflects patients’ metabolic status and disease severity (21). Several studies have established a relationship between elevated LAR and poor prognosis across various cancers, including gastrointestinal cancers (22–24), as well as other types such as breast and nasopharyngeal cancers (25, 26). However, further research is needed to fully understand the correlation between LAR and clinical outcomes in advanced NSCLC.

This dissertation aims to determine the prognostic value of pretreatment LAR in advanced NSCLC patients treated with first-line PD-1 checkpoint inhibitors combined with chemotherapy while controlling for other covariates.

## 2 Materials and methods

### 2.1 Patients

This study enrolled the patients with advanced NSCLC who received first-line systemic anti-PD-1 therapy plus chemotherapy at Guangxi Medical University Cancer Hospital between May 26th, 2019, and March 21st, 2023. To be included in this study, participants had to meet the following criteria: (1) A diagnosis of primary lung cancer confirmed by pathological examination by the third edition of the 2015 WHO classification of lung cancer; (2) Stage IV non-small cell lung cancer; (3) An Eastern Cooperative Oncology Group (ECOG) performance status score of 0-2 points; (4) Receiving PD-1 inhibitors combined with a platinum-based chemotherapy regimen for at least 2 cycles. Participants were excluded from this study if they met any of the following criteria: (1) Cases with no follow-up data or missing data; (2) Patients with active infections or inflammatory diseases; (3) Primary malignant tumors of other systems; (4) Patients treated with drugs other than PD-1 inhibitors combined with chemotherapy.

The final data analysis was conducted on 210 participants who met the inclusion and exclusion criteria. The PD-1 inhibitors including sintilimab, tislelizumab, pembrolizumab, camrelizumab and toripalimab were administrated intravenously every three weeks until disease progression or unacceptable toxicities.

### 2.2 Data collection

Clinicopathological characteristics of the patients, including LAR, age, sex, smoking history, tumor family history, ECOG-PS, histological type, PD-L1 Tumor Proportion Score (TPS), TNM stage, brain metastasis, liver metastasis, bone metastasis, adrenal metastasis, were extracted from medical records. Non-smokers are defined as patients who have smoked no more than 100 cigarettes in their lifetime. Individuals who had stopped smoking for < 1 year or who were current smokers before diagnosis are defined as smokers. The response of the tumor was evaluated using CT with Response Evaluation Criteria in Solid Tumors criteria (RECIST) version 1.1 every two cycles until treatment discontinuation or disease progression. Progression-free survival (PFS) was defined as the time from diagnosis to the date of the first documented event of tumor progression or death in the absence of disease progression. Overall survival (OS) was calculated from the date of diagnosis of advanced disease to the date of patient death or last follow-up.

Our data did not include identifiable patients’ data for the purpose of safeguarding patients’ privacy. The investigation complied with the ethical principles outlined in the Declaration of Helsinki. Participant informed consent was waived due to the retrospective nature of the study. The conduct of this study was authorized by the Ethics Committee of Guangxi Medical University Cancer Hospital (Approval number: LW202411).

### 2.3 Follow-up

We performed the follow-up through the telephone inquiry every three months. Follow-up data was documented and saved in the Hospital electronic medical record system. The cutoff date for follow-up was set at August 15th, 2023.

### 2.4 Statistical analysis

Continuous variables were expressed as mean ± standard deviation for normal distributions or median (min, max) for skewed distributions. Categorical variables were presented as a frequency or percentage. Differences among LAR groups were tested using χ2 for categorical variables, Student’s T-test for normal distributions, or Krusckal Wallis H test for skewed distributions. Fisher’s exact test was applied for comparisons in the study based on the theoretical frequency present in 2x2 table cells. For tables larger than 2x2, where more than 25% of cells had a frequency less than 5, the Freeman-Halton extension of Fisher’s test was used instead. The ROC curve was used to differentiate between low and high ratio groups, with an optimal LAR cutoff value of 5.0 identified. The impact of LAR on PFS and OS was assessed using Kaplan-Meier curves and the log-rank test. Subgroup analyses were conducted using stratified Cox proportional hazard models. The Cox proportional hazards regression model was employed for univariate and multivariate analyses to verify the independent predictive value of pretreated LAR in OS. Potential confounding covariates were adjusted in the multivariate analysis to ensure the accuracy and robustness of the results. Covariates were selected for adjustment if the effect estimate changed by more than 10% or the P value in the univariate analysis was less than 0.1. Statistical analyses were performed using R (version 3.4.3) and Empower Stats software. Statistical significance was determined by P values less than 0.05 (two-sided).

## 3 Results

### 3.1 Baseline characteristics of selected patients

The baseline characteristics of these selected patients are shown in **Table 1**, according to the Clinical cut point of LAR. Among the 210 participants chosen, the mean age was 58.56 ± 10.61 years old, with males making up approximately 79.05% of the group. Most patients (95.24%) have an ECOG-PS score of 0-1. TNM stage has statistical significance in both high and low groups (P value = 0.016). No significant differences were detected in age, sex, smoking history, tumor history, ECOG-PS, PD-L1 TPS, brain metastasis, liver metastasis, bone metastasis, pleural metastasis, adrenal metastasis, chemotherapy, immunotherapy among the different LAR groups, with all p values being more significant than 0.05.

**Table 1:** Baseline characteristics of advanced NSCLC patients.

Variable	N (%)	LAR <5.0	LAR ≥5.0	P value
Age				0.766
<65	141 (67.14%)	52 (68.42%)	89 (66.42%)	
≥65	69 (32.86%)	24 (31.58%)	45 (33.58%)	
Sex				0.979
Male	166 (79.05%)	60 (78.95%)	106 (79.10%)	
Female	44 (20.95%)	16 (21.05%)	28 (20.90%)	
Smoking history				0.84
Never	81 (38.57%)	30 (39.47%)	51 (38.06%)	
Ever	129 (61.43%)	46 (60.53%)	83 (61.94%)	
Tumor family history				0.818
No	173 (82.38%)	62 (81.58%)	111 (82.84%)	
Yes	37 (17.62%)	14 (18.42%)	23 (17.16%)	
ECOG-PS				0.077
0-1	200 (95.24%)	75 (98.68%)	125 (93.28%)	
2	10 (4.76%)	1 (1.32%)	9 (6.72%)	
Histological type				0.358
Adenocarcinoma	127 (60.48%)	50 (65.79%)	77 (57.46%)	
Squamous cell carcinoma	71 (33.81%)	21 (27.63%)	50 (37.31%)	
Other	12 (5.71%)	5 (6.58%)	7 (5.22%)	
TNM stage				0.016
IVA	115 (54.76%)	50 (65.79%)	65 (48.51%)	
IVB	95 (45.24%)	26 (34.21%)	69 (51.49%)	
Brain metastasis				0.367
No	182 (86.67%)	68 (89.47%)	114 (85.07%)	
Yes	28 (13.33%)	8 (10.53%)	20 (14.93%)	
Liver metastasis				0.158
No	175 (83.33%)	67 (88.16%)	108 (80.60%)	
Yes	35 (16.67%)	9 (11.84%)	26 (19.40%)	
Bone metastasis				0.095
No	128 (60.95%)	52 (68.42%)	76 (56.72%)	
Yes	82 (39.05%)	24 (31.58%)	58 (43.28%)	
Pleural metastasis				0.880
No	134 (63.81%)	49 (64.47%)	85 (63.43%)	
Yes	76 (36.19%)	27 (35.53%)	49 (36.57%)	
Adrenal metastasis				0.146
No	181 (86.19%)	69 (90.79%)	112 (83.58%)	
Yes	29 (13.81%)	7 (9.21%)	22 (16.42%)	
PD-L1 TPS				0.123
PD-L1<1%	26 (12.38%)	11 (14.47%)	15 (11.19%)	
PD-L1 ≥1%	56 (26.67%)	14 (18.42%)	42 (31.34%)	
PD-L1 1%-49%	30 (14.29%)	9 (11.84%)	21 (15.67%)	
PD-L1 ≥50%	16 (7.62%)	4 (5.26%)	12 (8.96%)	
Unknown	128 (60.95%)	51 (67.11%)	77 (57.46%)	
Chemotherapy				0.327
Pemetrexed	127 (60.48%)	51 (67.11%)	76 (56.72%)	
Taxanes	74 (35.24%)	22 (28.95%)	52 (38.81%)	
Gemcitabine	9 (4.29%)	3 (3.95%)	6 (4.48%)	
Immunotherapy				0.549
Sintilimab	54 (25.71%)	17 (22.37%)	37 (27.61%)	
Tislelizumab	103 (49.05%)	40 (52.63%)	63 (47.01%)	
Pabocilibab	12 (5.71%)	5 (6.58%)	7 (5.22%)	
Camrelizumab	20 (9.52%)	9 (11.84%)	11 (8.21%)	
Toripalimab	21 (10.00%)	5 (6.58%)	16 (11.94%)	

HR, hazard ratio; CI, confidence interval; ECOG-PS, Eastern Cooperative Oncology Group Performance Status; TNM, tumor, node, and metastases; PD-L1 TPS, PD-L1 Tumor Proportion Score; LAR, lactate dehydrogenase to albumin ratio.

### 3.2 Cutoff determination of LAR

**Figure 1:**
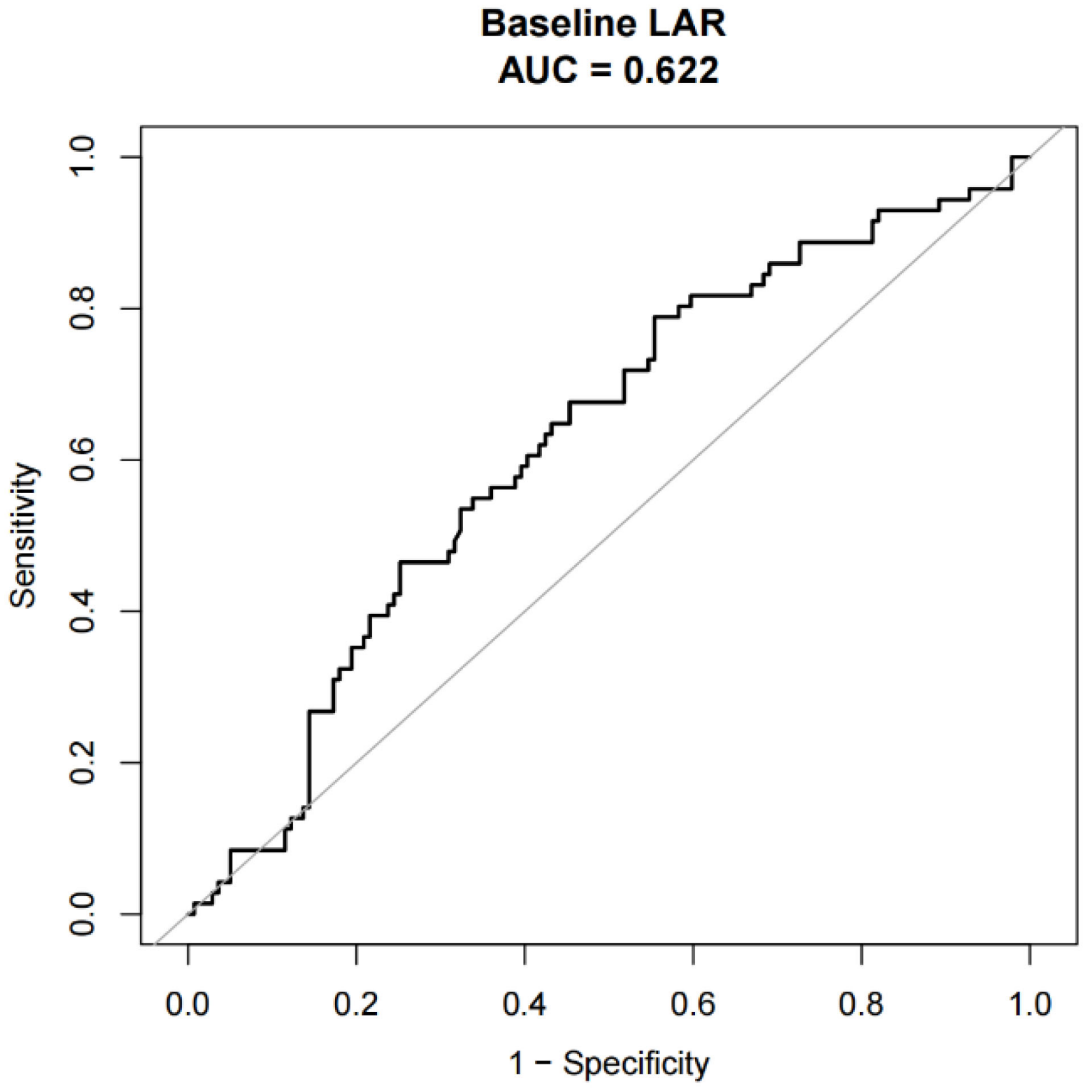
The determination of the optimal cutoff of pretreatment LAR. LAR, lactate dehydrogenase to albumin ratio.

The ROC curve was used to distinguish between the low and high ratio groups. The optimal cutoff value for LAR was 5.0, which resulted in a sensitivity of 78.87% and a specificity of 44.6% (area under the ROC curve, 0.622; P value = 0.001) (**Figure 1**). Based on this cutoff value, 76 patients (36.19%) had a lower LAR before treatment, and 134 patients (63.81%) had a higher LAR.

### 3.3 Confirmed objective response rate

In evaluating the treatment efficacy within the two patient groups, we noted an objective response rate (ORR) of 44.74% for the low LAR group and 53.73% for the high LAR group (**Table 2**). The disease control rate (DCR) was also closely aligned, with 90.79% for the low group and 89.55% for the high group. Although the ORR and DCR were slightly different in the two groups, the differences were not statistically significant with P values of 0.105 and 0.349, respectively. Notably, no patients in either group achieved a complete response.

**Table 2:** Summary of confirmed response assessed by RECIST Version 1.1.

Confirmed Response	LAR <5.0 N (%)	LAR ≥5.0 N (%)	P value
Best response			
PR	34(44.74%)	72 (53.73%)	
SD	35(46.05%)	48(35.82%)	
PD	6(7.90%)	6(4.48%)	
Not evaluable	1(1.32%)	8(5.97%)	
ORR	34(44.74%)	72 (53.73%)	0.105
DCR	69(90.79%)	120(89.55%)	0.349

Objective response rate(ORR) = Complete response (CR) + Partial response (PR); Disease control rate (DCR) = Complete response (CR) + Partial response (PR) + Stable disease (SD); Not evaluable = Patients who did not have one postbaseline imaging assessment; RECIST, Response Evaluation Criteria In Solid Tumors; LAR, lactate dehydrogenase to albumin ratio.

### 3.4 Results of univariates and multivariate analysis

The results of univariate and multivariate analyses of PFS can be found in **Table 3**. The univariate analysis yielded no correlation between age, sex, smoking history, tumor family history, ECOG-PS, histological type, adrenal metastasis, PD-L1 TPS, chemotherapy, immunotherapy, LAR, and PFS. In contrast, it showed that TNM stage, brain metastasis, liver metastasis, bone metastasis, and adrenal metastasis were positively correlated with PFS. In multivariate analysis, we adjust for confounding predictors and variables with a P-value less than 0.05 in the univariate analysis, including Age, Sex, Smoking history, ECOG-PS, TNM stage, Brain metastasis, Liver metastasis, Bone metastasis, Adrenal metastasis. We observed that brain metastasis, liver metastasis, and adrenal metastasis were significant prognostic variables (**Table 3**).

**Table 3:** Univariate and multivariate analyses of PFS.

Variable	N (%)	Univariate analysis		Multivariate analysis	
		HR (95%CI)	P value	HR (95% CI)	P value
Age					
<65	104 (67.53%)	1			
≥65	50 (32.47%)	0.90(0.59, 1.37)	0.619		
Sex					
Male	117 (75.97%)	1			
Female	37 (24.03%)	1.21(0.78, 1.87)	0.391		
Smoking history					
Never	66 (42.86%)	1			
Ever	88 (57.14%)	0.93(0.63, 1.36)	0.695		
Tumor family history					
No	128 (83.12%)	1			
Yes	26 (16.88%)	0.84(0.49, 1.44)	0.527		
ECOG-PS					
0-1	148 (96.10%)	1			
2	6 (3.90%)	1.73(0.63, 4.72)	0.288		
Histological type					
Adenocarcinoma	93 (60.39%)	1			
Squamous cell carcinoma	52 (33.77%)	0.97(0.64, 1.46)	0.887		
Other	9 (5.84%)	0.61(0.22, 1.69)	0.345		
TNM stage					
IVA	82 (53.25%)	1			
IVB	72 (46.75%)	1.76(1.19, 2.61)	0.005	1.26 (0.76, 2.09)	0.36
Brain metastasis					
No	135 (87.66%)	1			
Yes	19 (12.34%)	1.99(1.14, 3.45)	0.015	1.82 (1.00, 3.30)	0.049
Liver metastasis					
No	127 (82.47%)	1			
Yes	27 (17.53%)	1.89(1.18, 3.04)	0.009	1.87 (1.14, 3.09)	0.014
Bone metastasis					
No	94 (61.04%)	1			
Yes	60 (38.96%)	1.51(1.02, 2.22)	0.039	1.22 (0.76, 1.95)	0.414
Pleural metastasis					
No	103 (66.88%)	1			
Yes	51 (33.12%)	1.30(0.86, 1.96)	0.208		
Adrenal metastasis					
No	132 (85.71%)	1			
Yes	22 (14.29%)	1.73(1.03, 2.88)	0.037	1.81 (1.04, 3.16)	0.036
PD-L1 TPS					
PD-L1 <1%	22 (14.29%)	1			
PD-L1 ≥1%	48 (31.17%)	0.72(0.40, 1.29)	0.262		
PD-L1 1%-49%	25 (16.23%)	0.75(0.38, 1.48)	0.407		
PD-L1 ≥50%	14 (9.09%)	0.60(0.27, 1.34)	0.214		
Unknown	84 (54.55%)	0.69(0.40, 1.18)	0.172		
Chemotherapy					
Pemetrexed	93 (60.39%)	1			
Taxanes	54 (35.06%)	0.99(0.66, 1.50)	0.978		
Gemcitabine	7 (4.55%)	0.70(0.25, 1.92)	0.488		
Immunotherapy					
Sintilimab	40 (25.97%)	1			
Tislelizumab	85 (55.19%)	1.18(0.73, 1.88)	0.499		
Pabocilibab	8 (5.19%)	1.39(0.59, 3.24)	0.452		
Camrelizumab	13 (8.44%)	1.50(0.74, 3.02)	0.262		
Toripalimab	8 (5.19%)	1.29(0.58, 2.86)	0.528		
LAR					
LAR <5.0	56 (36.36%)	1			
LAR ≥5.0	98 (63.64%)	1.22(0.81, 1.82)	0.342	1.036 (0.67, 1.59)	0.871

HR, hazard ratio; CI, confidence interval; ECOG-PS, Eastern Cooperative Oncology Group Performance Status; TNM, tumor, node, and metastases; PD-L1 TPS, PD-L1 Tumor Proportion Score; LAR, lactate dehydrogenase to albumin ratio; PFS, progression-free survival.

Univariate and multivariate analyses of OS are listed in **Table 4**. Univariate analysis showed that Age, adrenal metastasis, PD-L1 TPS, chemotherapy, and immunotherapy were not statistically significant with OS. However, smoking history, ECOG-PS, and LAR were positively correlated with OS. The HR of death was increased by 139% in the high (LAR ≥ 5.0) group compared to that of the low (LAR < 5.0) group with 95% CI = 1.35, 4.23, P value = 0.003. Then we constructed the multivariate Cox proportional hazard model to analyze the independent effects of LAR on OS. We adjusted for potential confounders including Age, Sex, Smoking history, ECOG-PS. Compared with the low LAR group, the high LAR group was associated with an increased risk of death (HR = 2.22, 95% CI = 1.25-3.96); comparisons were statistically significant with P value < 0.01(**Table 4**).

**Table 4:** Univariate and multivariate analyses of OS.

Variable	N (%)	Univariate Analysis		Multivariate Analysis	
		HR (95%CI)	P value	HR (95%CI)	P value
Age					
<65	141 (67.14%)	1			
≥65	69 (32.86%)	1.40(0.87, 2.26)	0.17		
Sex					
Male	166 (79.05%)	1			
Female	44 (20.95%)	0.74(0.40, 1.34)	0.319		
Smoking history					
Never	81 (38.57%)	1			
Ever	129 (61.43%)	1.88(1.12, 3.15)	0.017	1.93 (0.94, 3.95)	0.072
Tumor family history					
No	173 (82.38%)	1			
Yes	37 (17.62%)	1.25(0.70, 2.25)	0.45		
ECOG-PS					
0-1	200 (95.24%)	1			
2	10 (4.76%)	2.66(1.15, 6.16)	0.022	1.86 (0.79, 4.38)	0.153
Histological type					
Adenocarcinoma	127 (60.48%)	1			
Squamous cell carcinoma	71 (33.81%)	0.88(0.54, 1.45)	0.627		
Other	12 (5.71%)	0.44(0.11, 1.83)	0.262		
TNM stage					
IVA	115 (54.76%)	1			
IVB	95 (45.24%)	1.52(0.95, 2.42)	0.08		
Brain metastasis					
No	182 (86.67%)	1			
Yes	28 (13.33%)	1.77(0.99, 3.19)	0.055		
Liver metastasis					
No	175 (83.33%)	1			
Yes	35 (16.67%)	1.10(0.59, 2.05)	0.76		
Bone metastasis					
No	128 (60.95%)	1			
Yes	82 (39.05%)	1.10(0.68, 1.77)	0.692		
Pleural metastasis					
No	134 (63.81%)	1			
Yes	76 (36.19%)	0.93(0.57, 1.53)	0.777		
Adrenal metastasis					
No	181 (86.19%)	1			
Yes	29 (13.81%)	1.50(0.79, 2.86)	0.217		
PD-L1 TPS					
PD-L1 <1%	26 (12.38%)	1			
PD-L1 ≥1%	56 (26.67%)	1.50(0.60, 3.75)	0.391		
PD-L1 1%-49%	30 (14.29%)	1.66(0.60, 4.59)	0.324		
PD-L1 ≥50%	16 (7.62%)	1.22(0.37, 4.00)	0.743		
Unknown	128 (60.95%)	1.45(0.62, 3.39)	0.395		
Chemotherapy					
Pemetrexed	127 (60.48%)	1			
Taxanes	74 (35.24%)	0.96(0.59, 1.58)	0.884		
Gemcitabine	9 (4.29%)	0.52 (0.13, 2.16)	0.372		
Immunotherapy					
Sintilimab	54 (25.71%)	1			
Tislelizumab	103 (49.05%)	1.19(0.66, 2.15)	0.566		
Pabocilibab	12 (5.71%)	1.08(0.39, 2.94)	0.887		
Camrelizumab	20 (9.52%)	1.89(0.90, 3.97)	0.091		
Toripalimab	21 (10.00%)	0.64(0.25, 1.63)	0.347		
LAR					
LAR <5.0	76 (36.19%)	1			
LAR ≥5.0	134 (63.81%)	2.39(1.35, 4.23)	0.003	2.22 (1.25, 3.96)	0.007

HR, hazard ratio; CI, confidence interval; ECOG-PS, Eastern Cooperative Oncology Group Performance Status; TNM, tumor, node, and metastases; PD-L1 TPS, PD-L1 Tumor Proportion Score; LAR, lactate dehydrogenase to albumin ratio; OS, overall survival.

### 3.5 Subgroup analysis

**Figure 2:**
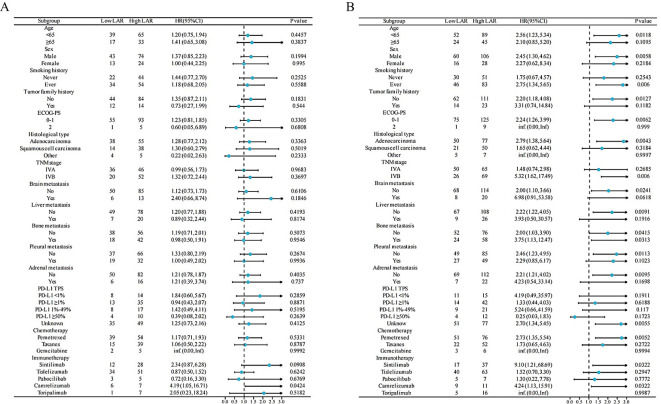
Forest plot of PFS **(A)** and OS **(B)** in the LAR group of NSCLC patients treated with PD-1 inhibitors and chemotherapy. HR, hazard ratio; CI, confidence interval; ECOG-PS, Eastern Cooperative Oncology Group Performance Status; TNM, tumor, node, and metastases; PD-L1 TPS, PD-L1 Tumor Proportion Score; LAR, lactate dehydrogenase to albumin ratio; PFS, progression-free survival; OS, overall survival.

We conducted stratified analysis to clarify the relationships between PFS (**Figure 2A**) and OS (**Figure 2B**) across different variables (**Supplementary Tables S1, S2**). The OS, instead of PFS, benefits in favor of Low LAR were observed across most subgroups (**Figure 2B**). We noted that patients with PD-L1 ≥ 50% had a 75% lower risk for death, but statistically, there is no significance (P value = 0. 172).

### 3.6 Kaplan–Meier survival analysis

**Figure 3:**
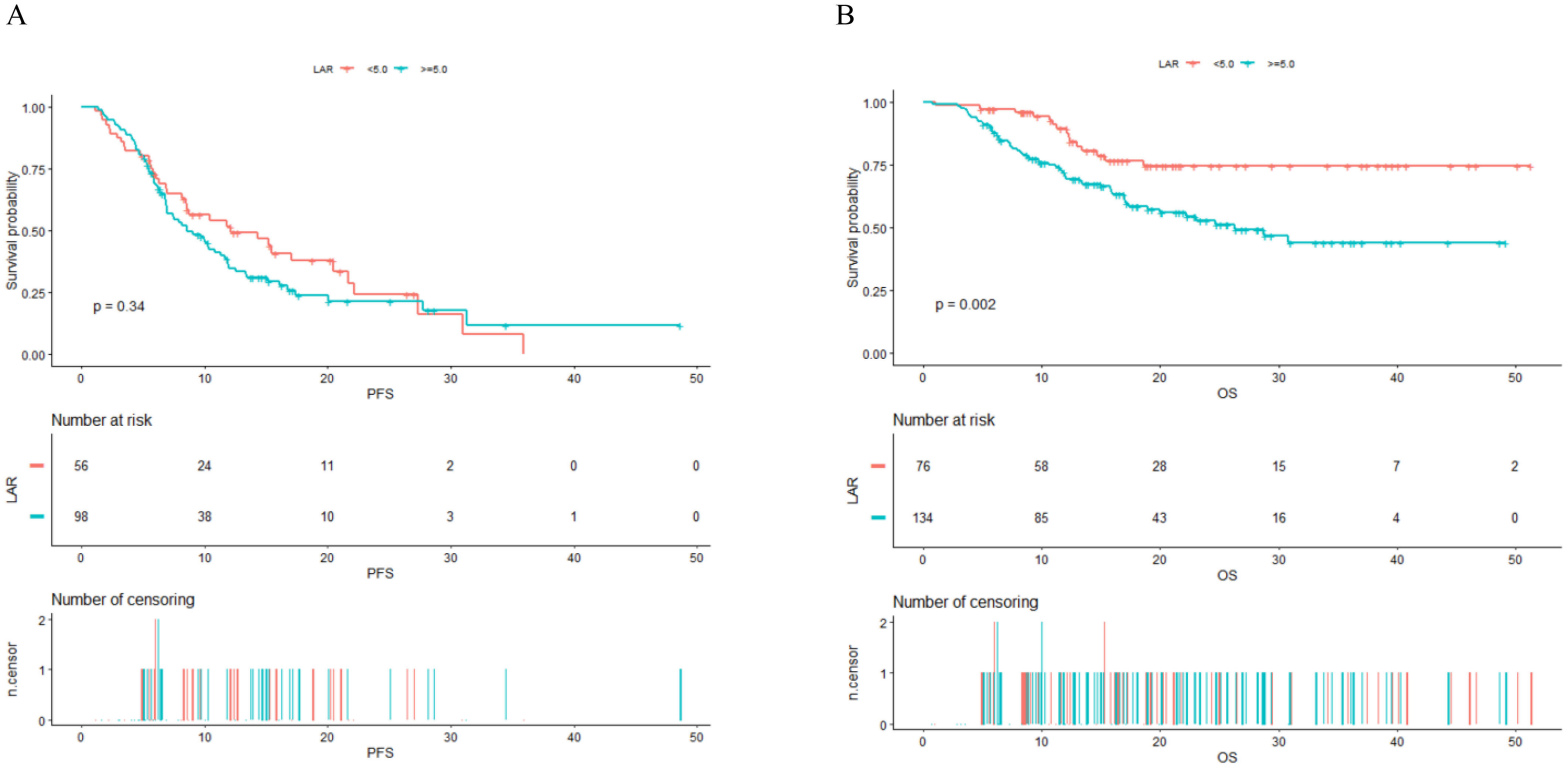
Kaplan–Meier survival analysis for PFS **(A)** and OS **(B)** in NSCLC patients treated with PD-1 checkpoint inhibitors and chemotherapy. PFS, progression-free survival; OS, overall survival; LAR, lactate dehydrogenase to albumin ratio.

**Figure 3** presents the Kaplan-Meier curves for PFS and OS, stratified by the two LAR groups. As depicted in **Figure 3A**, there is no significant statistical difference in PFS between the two groups (P value = 0.340). However, **Figure 3B** reveals a statistically significant difference in OS, with a P value of 0.002. The median OS for the high group (LAR ≥ 5.0) was 26.3 months. For the group with LAR < 5.0, the median OS has not yet been reached.

## 4 Discussion

In recent years, blood-based biomarkers, including bTMB, sPD-L1, sLAG-3 have been investigated as potential prognostic markers for patients with NSCLC treated with ICIs (27–29). However, due to the requirement of complex detection under specific conditions, which results in relatively high costs, they have not been widely recommended for routine use. Prognostic nutrition index (PNI) and C-reactive-protein-to-albumin ratio (CAR) are also valuable markers, and both are inherently linked to inflammation (30, 31). However, LAR focuses on the tumor itself and the overall systemic condition (32), demonstrating a relatively indirect association with inflammation. This provides a different perspective in assessing prognosis. Additionally, in many institutions in China, C-reactive protein (CRP) is not routinely tested and is measured primarily when inflammation is suspected, complicating the use of CAR as a research marker. PNI is highly variable due to its components, such as neutrophils, which have a short half-life of 6–12 hours (33), rendering it susceptible to fluctuations from inflammation and resulting in significant variations in PNI values when tested at different time points before treatment. In contrast, LDH and ALB are routinely tested markers for all patients before treatment. This makes the ratio easy to obtain, and it exhibit minimal short-term variability. In our study, we utilized the LAR to examine its prognostic value in patients with advanced NSCLC undergoing first-line anti-PD-1 inhibitors in conjunction with chemotherapy. Our findings revealed a negative association between LAR and OS. This could potentially provide critical information for clinicians, assisting in the assessment of disease status, the formulation of treatment strategies, as well as a more precise evaluation of patient prognosis.

LDH is an enzyme that aids in the transformation of lactate to pyruvate in cells. It plays a pivotal role in cancer cells due to their increased reliance on glycolysis - a process known as “the Warburg effect” (34). Instead of creating ATP through oxidative phosphorylation, these cells generate lactate through glycolytic metabolism, with LDH facilitating this lactate production (35). The result is an excess of lactate that acidifies the tumor microenvironment, which in turn fosters the survival and invasion of cancer cells (36). LDH’s role extends to managing the acid-base equilibrium within this microenvironment, thereby furthering tumor growth and dissemination (37). Research has highlighted direct correlations between elevated serum LDH levels and decreased survival rates in melanoma, prostate, and renal cell carcinomas (38, 39). This underlines the critical function of LDH in the progression of solid tumors.

ALB plays a critical role in patient nutrition, cancer progression, and immunity. As a crucial plasma protein, ALB contributes significantly to maintaining osmotic pressure and nutrient transport, including essential metals, hormones, and fatty acids, thereby supporting overall patient nutrition (40). Furthermore, ALB levels serve as a prognostic indicator in cancer progression (41, 42). Low ALB levels, known as hypoalbuminemia, are associated with poor prognosis in various cancers, indicating its role in cancer biology (43, 44). In the immune microenvironment, albumin can provide necessary nutritional and energy support for immune cells, thereby influencing their activity and function. Additionally, ALB’s role in immunity is underscored by its antioxidant and anti-inflammatory properties, as well as its involvement in lymphocyte function and cytokine transport (45). Hence, understanding albumin’s multifaceted roles can aid in patient management and therapeutic strategies.

The LAR, which integrates the tumor burden information supplied by LDH and the nutritional status indicated by ALB, offers a holistic view of a patient’s condition. This may serve as a more reliable prognostic indicator in cancer patients compared to LDH or ALB alone. Studies have concluded that the LAR could serve as a new prognostic factor for various cancers. Feng et al. discovered that high LAR levels were linked to poor Cancer-Specific Survival (CSS) in esophageal squamous cell carcinoma patients who underwent curative surgery without neoadjuvant therapy (46). Similarly, Ulaş Aday demonstrated that a high LAR could negatively impact prognosis in colorectal cancer patients after curative resection (24). Expanding on this, Xie et al. found a significant association between high LAR and worse PFS and OS (47). He et al. proposed that high preoperative LAR values could be an independent poor prognosis indicator for breast cancer (25). However, there have been few studies investigating LAR in advanced NSCLC with anti-PD-1 therapy. In this context, Menekse, S. et al. identified a strong correlation between LAR and both PFS and OS, suggesting that LAR could be a poor prognostic predictor for patients with NSCLC treated with nivolumab (48). Our study reveals that the LAR reliably as a prognosticator for OS; however, its utility in forecasting PFS appears to be limited. This discrepancy could potentially stem from the inherent characteristics of ICIs, notably their delayed action onset and pronounced long-term effects.

Prior researches have demonstrated a range of optimal cut-off values for the LAR in predicting survival outcomes. Two primary reasons account for this variability: First, the types of tumors under study differ significantly, and even within studies of the same tumor type, variations in sample size and subject selection can occur. Second, researchers often use diverse methodologies to determine the optimal cut-off value. For example, Feng used the X-tile program to establish an optimal LAR cut-off value of 5.5 (46). Conversely, Peng employed ROC curves to derive an optimal cut-off value of 4.04 (26), and Xie Z used R software to determine a cut-off value of 4.91 (47). Ulaş Aday performed a time-dependent ROC curve analysis to yield an optimal pretreatment Lactate Dehydrogenase-to-Albumin Ratio cut-off value of 52.7 (24). Simultaneously, Menekse, S.’s research employed ROC analysis to ascertain the optimal cut-off value (48). Our study also utilized the ROC curve to differentiate between groups with low and high ratios. We conjecture that these divergent optimal cut-off values may be linked to variations in sample sizes and the racial composition of the study populations.

Our study observed that a PD-L1 Tumor Proportion Score (TPS) ≥50% might potentially act as a protective factor against disease progression or mortality. However, this result did not reach statistical significance (P = 0.172), highlighting the complexity of using PD-L1 as a biomarker. The evaluation of PD-L1 expression is predominantly dependent on PD L1 scoring systems, notably TPS and Combined Positive Score (CPS), which evaluating expression on tumor and immune cells (49). Therefore, addressing the factors influencing these scorings is essential. Interobserver variability poses a significant challenge, as pathologists’ interpretations can differ, leading to inconsistent PD-L1 scoring (50). Standardized protocols and training can mitigate this variability. The choice of antibody clones, such as 22C3, SP142, and SP263, complicates assessments due to differing binding affinities (49). Furthermore, sample type selection plays a critical role in result consistency, with resections often yielding more reliable PD-L1 evaluation outcomes than biopsies (51). Tumor heterogeneity further complicates assessments, as PD-L1 expression can vary within and between tumors (51). In addition, differences in the sensitivity and specificity of diagnostic platforms challenge scoring consistency (52). Emerging technologies, such as artificial intelligence and computer image analysis, show promise in enhancing the accuracy and efficiency of evaluations (50, 53). These technologies offer tools to reduce variability and improve PD-L1 scoring precision, which is crucial for appropriate immunotherapy selection. Further validation with external data is essential to deepen our understanding of PD-L1’s multifaceted role as a biomarker in treatment decisions.

Our study exhibits several strengths: (1) We first utilized the LAR ratio to predict the prognosis of advanced non-small cell lung cancer patients in the Asian population receiving first-line immunotherapy combined with chemotherapy; (2) As an observational study, it is inherently prone to potential confounding. However, we employed rigorous statistical adjustment to mitigate the impact of residual confounders; (3) We processed the target independent variable both as a continuous and categorical variable. This approach decreases the contingency in data analysis and bolsters the robustness of our results; (4) A subgroup analysis was conducted to validate our robust and stable findings specifically within distinct subgroups.

Despite the valuable insights provided by our study, it is important to acknowledge some limitations. Firstly, our research is a single-center retrospective cohort study, which might introduce selection bias and possibly distort the observed association. Secondly, our focus was on the pretreatment LAR, and we did not analyze the dynamic changes during the tumor progression process. Thirdly, in the clinicopathological characteristics of this study, the ratio of men to women is not balanced enough. More than half of Asian women carry common driver gene mutations and are often suitable for targeted therapy in the first-line setting, which results in a relatively high proportion of men in our study. This is also consistent with the population characteristics of some large-scale RCT studies conducted in China, such as certain clinical studies (54). A larger scale, prospective validation study is required to confirm the generalizability of these results.

## 5 Conclusion

In conclusion, the pretreatment LAR might serve as a potential independent prognostic marker for patients with advanced NSCLC receiving a combination of PD-1 inhibitors and chemotherapy. However, to confirm the universality of these findings, a large-scale, prospective validation study is required.

## Data Availability

The original contributions presented in the study are included in the article/**Supplementary Material**. Further inquiries can be directed to the corresponding authors.
