# Supplementary material for: Prognostic value of the lactate dehydrogenase to albumin ratio in advanced non-small cell lung cancer patients treated with the first-line PD-1 checkpoint inhibitors combined with chemotherapy
Source: Front Immunol. 2025 Feb 12;16:1473962. doi: 10.3389/fimmu.2025.1473962 (PMC11861202; doi:10.3389/fimmu.2025.1473962)
Supplement: Supplementary file 1 [file Table1.docx]

**TABLES 1** The hierarchical analysis of PFS.

| Variable | N (%) | LAR <5.0 | LAR ≥5.0 | HR (95%CI) | P value |
| --- | --- | --- | --- | --- | --- |
| Age |  |  |  |  |  |
| <65 | 104(67.53%) | 39(69.64%) | 65 (66.33%) | 1.20 (0.75, 1.94) | 0.446 |
| ≥65 | 50 (32.47%) | 17(30.36%) | 33 (33.67%) | 1.41 (0.65, 3.08) | 0.384 |
| Sex |  |  |  |  |  |
| Male | 117 (75.97%) | 43 (76.79%) | 74 (75.51%) | 1.37 (0.85, 2.23) | 0.199 |
| Female | 37 (24.03%) | 13 (23.21%) | 24 (24.49%) | 1.00 (0.44, 2.25) | 0.995 |
| Smoking history |  |  |  |  |  |
| Never | 66(42.86%) | 22 (39.29%) | 44 (44.90%) | 1.44 (0.77, 2.70) | 0.253 |
| Ever | 88(57.14%) | 34 (60.71%) | 54 (55.10%) | 1.18 (0.68, 2.05) | 0.559 |
| Tumor family history |  |  |  |  |  |
| No | 128(83.12%) | 44 (78.57%) | 84 (85.71%) | 1.35 (0.87, 2.11) | 0.183 |
| Yes | 26(16.88%) | 12 (21.43%) | 14 (14.29%) | 0.73 (0.27, 1.99) | 0.544 |
| ECOG-PS |  |  |  |  |  |
| 0-1 | 148(96.10%) | 55 (98.21%) | 93 (94.90%) | 1.23 (0.81, 1.85) | 0.331 |
| 2 | 6(3.90%) | 1 (1.79%) | 5 (5.10%) | 0.60 (0.05, 6.89) | 0.681 |
| Histological type |  |  |  |  |  |
| Adenocarcinoma | 93(60.39%) | 38 (67.86%) | 55 (56.12%) | 1.28 (0.77, 2.12) | 0.336 |
| Squamous cell carcinoma | 52(33.77%) | 14 (25.00%) | 38 (38.78%) | 1.30 (0.60, 2.79) | 0.502 |
| Other | 9(5.84%) | 4 (7.14%) | 5 (5.10%) | 0.22 (0.02, 2.63) | 0.233 |
| TNM stage |  |  |  |  |  |
| IVA | 82 (53.25%) | 36 (64.29%) | 46 (46.94%) | 0.99 (0.56, 1.73) | 0.968 |
| IVB | 72 (46.75%) | 20 (35.71%) | 52 (53.06%) | 1.32 (0.72, 2.44) | 0.370 |
| Brain metastasis |  |  |  |  |  |
| No | 135(87.66%) | 50 (89.29%) | 85 (86.73%) | 1.12 (0.73, 1.73) | 0.611 |
| Yes | 19(12.34%) | 6 (10.71%) | 13 (13.27%) | 2.40 (0.66, 8.74) | 0.185 |
| Liver metastasis |  |  |  |  |  |
| No | 127(82.47%) | 49 (87.50%) | 78 (79.59%) | 1.20 (0.77, 1.88) | 0.419 |
| Yes | 27 (17.53%) | 7 (12.50%) | 20 (20.41%) | 0.89 (0.32, 2.44) | 0.817 |
| Bone metastasis |  |  |  |  |  |
| No | 94 (61.04%) | 38 (67.86%) | 56 (57.14%) | 1.19 (0.71, 2.01) | 0.507 |
| Yes | 60(38.96%) | 18 (32.14%) | 42 (42.86%) | 0.98 (0.50, 1.91) | 0.955 |
| Pleural metastasis |  |  |  |  |  |
| No | 103(66.88%) | 37 (66.07%) | 66 (67.35%) | 1.33 (0.80, 2.19) | 0.267 |
| Yes | 51 (33.12%) | 19 (33.93%) | 32 (32.65%) | 1.00 (0.49, 2.02) | 0.994 |
| Adrenal metastasis |  |  |  |  |  |
| No | 132(85.71%) | 50 (89.29%) | 82 (83.67%) | 1.21 (0.78, 1.87) | 0.404 |
| Yes | 22(14.29%) | 6 (10.71%) | 16 (16.33%) | 1.21 (0.39, 3.74) | 0.737 |
| PD-L1 TPS |  |  |  |  |  |
| PD-L1 <1% | 22(14.29%) | 8 (14.29%) | 14 (14.29%) | 1.84 (0.60, 5.67) | 0.286 |
| PD-L1 ≥1% | 48(31.17%) | 13 (23.21%) | 35 (35.71%) | 0.94 (0.43, 2.07) | 0.887 |
| PD-L1 1%-49% | 25(16.23%) | 8 (14.29%) | 17 (17.35%) | 1.42 (0.49, 4.11) | 0.520 |
| PD-L1 ≥50% | 14 (9.09%) | 4 (7.14%) | 10 (10.20%) | 0.39 (0.08, 2.02) | 0.264 |
| Unknown | 84(54.55%) | 35 (62.50%) | 49 (50.00%) | 1.25 (0.73, 2.16) | 0.413 |
| Chemotherapy |  |  |  |  |  |
| Pemetrexed | 93(60.39%) | 39 (69.64%) | 54 (55.10%) | 1.17 (0.71, 1.93) | 0.533 |
| Taxanes | 54(35.06%) | 15 (26.79%) | 39 (39.80%) | 1.06 (0.50, 2.22) | 0.879 |
| Gemcitabine | 7(4.55%) | 2 (3.57%) | 5 (5.10%) | inf. (0.00, Inf) | 0.999 |
| Immunotherapy |  |  |  |  |  |
| Sintilimab | 40 (25.97%) | 12 (21.43%) | 28 (28.57%) | 2.34 (0.87, 6.28) | 0.091 |
| Tislelizumab | 85(55.19%) | 34 (60.71%) | 51 (52.04%) | 0.87 (0.50, 1.52) | 0.624 |
| Pabocilibab | 8 (5.19%) | 3 (5.36%) | 5 (5.10%) | 0.72 (0.16, 3.30) | 0.677 |
| Camrelizumab | 13(8.44%) | 6 (10.71%) | 7 (7.14%) | 4.19 (1.05, 16.71) | 0.042 |
| Toripalimab | 8(5.19%) | 1 (1.79%) | 7 (7.14%) | 2.05 (0.23, 18.24) | 0.518 |

Abbreviations: HR, hazard ratio; CI, confidence interval; ECOG-PS, Eastern Cooperative Oncology Group Performance Status; TNM, tumor, node, and metastases; PD-L1 TPS, PD-L1 Tumor Proportion Score; LAR, lactate dehydrogenase to albumin ratio; PFS, progression-free survival.

**TABLES 2** The hierarchical analysis of OS.

| Variable | N (%) | LAR <5.0 | LAR ≥5.0 | HR (95%CI) | P value |
| --- | --- | --- | --- | --- | --- |
| Age |  |  |  |  |  |
| <65 | 141 (67.14%) | 52 (68.42%) | 89 (66.42%) | 2.56 (1.23, 5.34) | 0.012 |
| ≥65 | 69 (32.86%) | 24 (31.58%) | 45 (33.58%) | 2.10 (0.85, 5.20) | 0.110 |
| Sex |  |  |  |  |  |
| Male | 166(79.05%) | 60 (78.95%) | 106 (79.10%) | 2.45 (1.30, 4.62) | 0.006 |
| Female | 44(20.95%) | 16 (21.05%) | 28 (20.90%) | 2.27 (0.62, 8.34) | 0.218 |
| Smoking history |  |  |  |  |  |
| Never | 81 (38.57%) | 30 (39.47%) | 51 (38.06%) | 1.75 (0.67, 4.57) | 0.254 |
| Ever | 129 (61.43%) | 46 (60.53%) | 83 (61.94%) | 2.75 (1.34, 5.65) | 0.006 |
| Tumor family history | |  |  |  |  |
| No | 173 (82.38%) | 62 (81.58%) | 111 (82.84%) | 2.20 (1.18, 4.08) | 0.013 |
| Yes | 37 (17.62%) | 14 (18.42%) | 23 (17.16%) | 3.31 (0.74, 14.84) | 0.118 |
| ECOG-PS |  |  |  |  |  |
| 0-1 | 200(95.24%) | 75 (98.68%) | 125 (93.28%) | 2.24 (1.26, 3.99) | 0.006 |
| 2 | 10(4.76%) | 1 (1.32%) | 9 (6.72%) | inf. (0.00, Inf) | 0.999 |
| Histological type |  |  |  |  |  |
| Adenocarcinoma | 127(60.48%) | 50 (65.79%) | 77 (57.46%) | 2.79 (1.38, 5.64) | 0.004 |
| Squamous cell carcinoma | 71 (33.81%) | 21 (27.63%) | 50 (37.31%) | 1.65 (0.62, 4.44) | 0.318 |
| Other | 12 (5.71%) | 5 (6.58%) | 7 (5.22%) | inf. (0.00, Inf) | 1.000 |
| TNM stage |  |  |  |  |  |
| IVA | 115(54.76%) | 50 (65.79%) | 65 (48.51%) | 1.48 (0.74, 2.98) | 0.269 |
| IVB | 95 (45.24%) | 26 (34.21%) | 69 (51.49%) | 5.32 (1.62, 17.49) | 0.006 |
| Brain metastasis |  |  |  |  |  |
| No | 182(86.67%) | 68 (89.47%) | 114 (85.07%) | 2.00 (1.10, 3.66) | 0.024 |
| Yes | 28 (13.33%) | 8 (10.53%) | 20 (14.93%) | 6.98 (0.91, 53.58) | 0.062 |
| Liver metastasis |  |  |  |  |  |
| No | 175(83.33%) | 67 (88.16%) | 108 (80.60%) | 2.22 (1.22, 4.05) | 0.009 |
| Yes | 35(16.67%) | 9 (11.84%) | 26 (19.40%) | 3.93 (0.50, 30.57) | 0.192 |
| Bone metastasis |  |  |  |  |  |
| No | 128 (60.95%) | 52 (68.42%) | 76 (56.72%) | 2.00 (1.03, 3.90) | 0.042 |
| Yes | 82(39.05%) | 24 (31.58%) | 58 (43.28%) | 3.75 (1.13, 12.47) | 0.031 |
| Pleural metastasis |  |  |  |  |  |
| No | 134(63.81%) | 49 (64.47%) | 85 (63.43%) | 2.46 (1.23, 4.95) | 0.011 |
| Yes | 76(36.19%) | 27 (35.53%) | 49 (36.57%) | 2.29 (0.85, 6.17) | 0.102 |
| Adrenal metastasis |  |  |  |  |  |
| No | 181 (86.19%) | 69 (90.79%) | 112 (83.58%) | 2.21 (1.21, 4.02) | 0.010 |
| Yes | 29(13.81%) | 7 (9.21%) | 22 (16.42%) | 4.23 (0.54, 33.14) | 0.170 |
| PD-L1 TPS |  |  |  |  |  |
| PD-L1 <1% | 26(12.38%) | 11 (14.47%) | 15 (11.19%) | 4.19 (0.49, 35.97) | 0.191 |
| PD-L1 ≥1% | 56(26.67%) | 14 (18.42%) | 42 (31.34%) | 1.33 (0.44, 4.03) | 0.619 |
| PD-L1 1%-49% | 30(14.29%) | 9 (11.84%) | 21 (15.67%) | 5.24 (0.66, 41.59) | 0.117 |
| PD-L1 ≥50% | 16(7.62%) | 4 (5.26%) | 12 (8.96%) | 0.25 (0.03, 1.83) | 0.172 |
| Unknown | 128(60.95%) | 51 (67.11%) | 77 (57.46%) | 2.70 (1.34, 5.45) | 0.006 |
| Chemotherapy |  |  |  |  |  |
| Pemetrexed | 127(60.48%) | 51 (67.11%) | 76 (56.72%) | 2.73 (1.35, 5.54) | 0.005 |
| Taxanes | 74(35.24%) | 22 (28.95%) | 52 (38.81%) | 1.73 (0.65, 4.63) | 0.272 |
| Gemcitabine | 9(4.29%) | 3 (3.95%) | 6 (4.48%) | inf. (0.00, Inf) | 0.999 |
| Immunotherapy |  |  |  |  |  |
| Sintilimab | 54(25.71%) | 17 (22.37%) | 37 (27.61%) | 9.10 (1.21, 68.69) | 0.032 |
| Tislelizumab | 103 (49.05%) | 40 (52.63%) | 63 (47.01%) | 1.52 (0.70, 3.30) | 0.295 |
| Pabocilibab | 12(5.71%) | 5 (6.58%) | 7 (5.22%) | 1.30 (0.22, 7.78) | 0.777 |
| Camrelizumab | 20 (9.52%) | 9 (11.84%) | 11 (8.21%) | 4.24 (1.13, 15.91) | 0.032 |
| Toripalimab | 21 (10.00%) | 5 (6.58%) | 16 (11.94%) | inf. (0.00, Inf) | 0.999 |

Abbreviations: HR, hazard ratio; CI, confidence interval; ECOG-PS, Eastern Cooperative Oncology Group Performance Status; TNM, tumor, node, and metastases; PD-L1 TPS, PD-L1 Tumor Proportion Score; LAR, lactate dehydrogenase to albumin ratio; OS, overall survival.
